# Differences in the prevalence of methicillin-resistant *Staphylococcus aureus* (MRSA) among health-care workers using a “single” vs. “double” screening strategy

**DOI:** 10.1186/1753-6561-5-S6-P15

**Published:** 2011-06-29

**Authors:** R Saßmannshausen, R Köck, A Jurke, AW Friedrich

**Affiliations:** 1 Institute of Hygiene, University Hospital Muenster, Muenster, Germany; 2 NRW Institute of Health and Work, Muenster, Germany; 3 Dept. for Medical Microbiologie and Infection Control, University Hospital Groningen, Groningen, Netherlands

## Introduction / objectives

Within healthcare institutions, healthcare workers (HCW) who are nasal MRSA carriers can facilitate its spread. This study assesses the colonization rate of HCW outside outbreak situations and compares results of a “single” vs. “double” strategy of MRSA-screening.

## Methods

HCW from nine hospitals in the German part of the Dutch-German EUREGIO participated. Two nasal-pharyngeal swabs were derived from each participant on two days. MRSA were characterized using typing of the *S. aureus*protein A gene (*spa*). If MRSA was detected from both specimens, a decolonization therapy was used.

## Results

726 HCW have participated in the study.33 participants (4.5%) were MRSA-positive in at least one swab. 23 of them (3.2%) were positive in both swabs, whereas for 10 persons (30% of all participants with at least one positive result) MRSA was isolated on one of the two days. Among physicians, nurses and other personnel, the prevalence of MRSA was 1.2%, 3.7% and 2.8%, respectively. Nine different *spa*-types were detected with typical hospital-acquired strains (t003, t032) being the most frequent.

## Conclusion

The study revealed that outside outbreaks situations, HCW are frequently colonized with typical hospital-acquired MRSA clones. Notably, livestock-associated MRSA molecular types were not found in this study although such strains are amongst the predominant MRSA lineages isolated from patients. If HCW were tested on a single day, the prevalence was 30% lower compared to a duplicate testing. This might either reflect the less than 100% sensitivity of nasopharyngeal screening cultures or a non-persistent MRSA carriage. Importantly, all HCW were successfully decolonized.

## Disclosure of interest

None declared.

